# Multimodal Sensor Fusion in Autonomous Vehicles: Technologies, Architectures, and Open Challenges

**DOI:** 10.3390/s26113528

**Published:** 2026-06-02

**Authors:** Patrik Viktor, Gabor Kiss

**Affiliations:** 1 Keleti Károly Faculty of Business and Management, Obuda University, 1034 Budapest, Hungary; viktor.patrik@uni-obuda.hu; 2 Institute of Safety Science and Cybersecurity, Obuda University, 1034 Budapest, Hungary; 3 Department of Computer Science, J. Selye University, 945 01 Komarno, Slovakia

**Keywords:** autonomous vehicles, multimodal sensor fusion, LiDAR, radar, transformer-based fusion, Bird’s Eye View (BEV), functional safety, uncertainty-aware perception, edge AI

## Abstract

The rapid progress of sensing technologies, artificial intelligence, and embedded computing has significantly accelerated the development of autonomous vehicles. Among the core challenges of higher-level driving automation, reliable environmental perception remains one of the most critical. This review presents a systematic PRISMA-based analysis of multimodal sensor technologies and fusion architectures applied in autonomous driving, based on 66 peer-reviewed studies published between 2014 and 2025. The study examines the operational characteristics, advantages, and limitations of major sensing modalities, including cameras, LiDAR, radar, ultrasonic sensors, and GNSS/IMU-based localization systems. Particular attention is given to multimodal fusion strategies, covering early, mid-level, high-level, and transformer-based architectures that combine complementary sensor information to improve perception robustness and decision reliability. The review further synthesizes current evidence on performance under adverse environmental conditions, benchmark validation practices, real-time computational constraints, and the growing role of functional safety frameworks such as ISO 26262 and SOTIF. Emerging research directions, including 4D radar, self-supervised long-range fusion, foundation models, and cooperative V2X perception, are also discussed. The findings indicate that multimodal sensor fusion is a highly effective architectural strategy for improving scalability, fail-operational robustness, and certifiable safety in autonomous driving systems, particularly in higher-level automation scenarios. Future research should focus on uncertainty-aware fusion, explainable cross-modal reasoning, large-scale real-world validation, and efficient hardware–software co-design to support robust Level 4–5 vehicle autonomy.

## 1. Introduction

Autonomous driving has the potential to fundamentally transform mobility by improving road safety, reducing congestion, and increasing accessibility. As automation levels advance toward SAE Level 4–5 systems, reliable environmental perception becomes a critical prerequisite for safe and scalable deployment [1]. Because no single sensing modality can provide robust scene understanding across all operational conditions, modern autonomous vehicles rely on multimodal perception stacks that combine cameras, LiDAR, radar, ultrasonic sensors, and GNSS/IMU-based localization systems [2].

Each sensing technology contributes distinct strengths and limitations. Cameras provide rich semantic and texture information, LiDAR delivers precise three-dimensional geometry, and radar offers robust range and velocity estimation under adverse weather and low-visibility conditions [3,4]. The complementary nature of these modalities makes multimodal sensor fusion a frequently preferred architectural strategy, particularly when robustness, redundancy, and fail-operational behavior are prioritized over minimal system complexity. By integrating redundant and complementary information, fusion architectures improve detection reliability, fault tolerance, and operational safety in complex real-world environments.

Recent years have seen rapid advances in deep learning-based perception, transformer architectures, 4D imaging radar, self-supervised long-range fusion, and safety-aware fail-operational system design. However, the literature remains fragmented across sensing technologies, fusion strategies, benchmark evaluation, robustness studies, and functional safety frameworks. Existing reviews often focus on individual sensor modalities or specific algorithmic paradigms, with limited attention to the interaction between perception performance, environmental robustness, computational constraints, and certifiable automotive safety. To address this gap, the present review provides a systematic synthesis of multimodal sensor technologies, fusion architectures, validation benchmarks, robustness challenges, and emerging research directions in autonomous driving. Particular emphasis is placed on the relationship between sensor complementarity, fusion design choices, adverse-weather resilience, and safety-oriented fail-operational perception architectures aligned with ISO 26262 and SOTIF. The objective is to provide an integrated technical reference that supports both future research and practical system development in autonomous vehicle perception [4].

Beyond synthesizing existing literature, the core innovation of this review lies in its explicit integration of multimodal sensing, fusion architectures, robustness evaluation, and functional safety considerations within a unified systems-level framework. Unlike prior surveys that treat these dimensions in isolation, this work systematically links perception performance with real-world deployment constraints, fail-operational requirements, and certifiable safety standards. This integrative perspective enables a deeper understanding of not only how multimodal fusion methods perform, but why certain architectural choices are more suitable for scalable and safety-critical autonomous driving systems [5].

### Related Reviews and Positioning of This Work

Several review articles have previously examined autonomous vehicle perception, sensor technologies, and deep learning–based fusion frameworks. Existing surveys typically focus on individual sensing modalities, such as LiDAR camera fusion, radar perception, or benchmark-specific deep learning architectures. More recent reviews have also discussed Bird’s Eye View (BEV) perception and transformer-based multimodal learning. However, these studies often emphasize algorithmic performance while providing limited discussion of environmental robustness, deployment constraints, and automotive safety validation.

The present review extends prior work in four important directions. First, it provides a unified synthesis across the full multimodal sensing stack, including cameras, LiDAR, radar, ultrasonic sensing, GNSS/IMU localization, and emerging modalities such as event-based cameras and 4D imaging radar. Second, it integrates classical and modern fusion taxonomies, covering early, mid-level, high-level, and transformer-based architectures within a single comparative framework. Third, unlike many earlier reviews, this study explicitly connects perception performance with robustness under adverse weather, uncertainty-aware fusion, and real-time edge deployment constraints. Fourth, particular emphasis is placed on functional safety and fail-operational architectures, linking multimodal fusion design to ISO 26262 and SOTIF requirements. By combining sensor physics, fusion architectures, validation benchmarks, robustness analysis, computational deployment, and certifiable safety considerations, this review aims to provide a broader systems-engineering perspective than prior surveys. This positioning is particularly important given the rapid emergence of 4D radar, self-supervised long-range perception, foundation models, and cooperative V2X sensing, which are reshaping the design space of autonomous vehicle perception systems.

This review differs from existing surveys by adopting a holistic systems-engineering perspective that explicitly integrates sensing technologies, fusion architectures, robustness evaluation, computational constraints, and functional safety considerations. In contrast to prior reviews that primarily emphasize algorithmic performance or individual modalities, this work connects these dimensions within a unified analytical framework. As a result, it provides a more deployment-oriented interpretation of multimodal fusion, highlighting not only performance characteristics but also implications for robustness, fail-operational behavior, and certifiable safety in real-world autonomous driving systems.

To improve the structural coherence of this review, the paper follows a systems-oriented analytical framework that connects sensing modalities, fusion architectures, perception functions, validation practices, and deployment constraints in a sequential logic. First, the physical sensing layer is examined by analyzing the operational principles, strengths, and limitations of individual sensor modalities. Second, the study maps how these complementary sensor characteristics motivate different fusion architectures, ranging from early and mid-level fusion to high-level probabilistic and transformer-based approaches. Third, the review links these architectural choices to downstream environmental perception tasks, including detection, segmentation, tracking, localization, and free-space estimation. Fourth, benchmark datasets, evaluation metrics, and adverse-condition testing strategies are synthesized to assess real-world robustness and reproducibility. Finally, the framework extends toward functional safety, computational deployment, and emerging research directions, thereby providing an end-to-end systems perspective on multimodal perception in autonomous vehicles.

## 2. Material and Methods

This review was conducted using a systematic literature review (SLR) methodology in accordance with the PRISMA (Preferred Reporting Items for Systematic Reviews and Meta-Analyses) framework. The adoption of PRISMA ensures methodological transparency, reproducibility, and structured reporting of the literature identification, screening, eligibility assessment, and inclusion process. Given the rapid evolution and multidisciplinary nature of multimodal sensor fusion in autonomous vehicles spanning robotics, computer vision, embedded systems, safety engineering, and artificial intelligencea systematic approach was essential to avoid selection bias and to provide a comprehensive synthesis of current knowledge. The review protocol was defined prior to initiating the search process in order to reduce methodological drift and ensure consistency. The protocol specified the research objectives, databases to be searched, search keywords and Boolean expressions, inclusion and exclusion criteria, screening procedures, data extraction categories, and quality assessment methodology. Establishing this protocol in advance enabled a structured and reproducible review process aligned with PRISMA 2020 reporting recommendations.

The primary aim of this review was to systematically analyze the technological foundations, architectural paradigms, validation practices, and emerging trends in multimodal sensor fusion for autonomous vehicles. To operationalize this aim, the following research questions were defined:

**RQ1:** Which sensor modalities are most frequently combined in autonomous vehicle perception systems?

**RQ2:** What fusion architectures are employed (early, mid-level, late, and deep learning-based fusion)?

**RQ3:** How is robustness under adverse environmental conditions evaluated and addressed?

**RQ4:** Which benchmark validation environments and evaluation metrics are most frequently reported across the included studies?

**RQ5:** How are computational constraints, safety standards, and functional validation incorporated into sensor fusion research?

These research questions guided the design of the search strategy and the categorization of extracted data.

### 2.1. Search Strategy and Information Sources

A comprehensive and systematic search was conducted across multiple leading scientific databases to ensure broad coverage of peer-reviewed research in engineering, robotics, and artificial intelligence. The following databases were included:

- IEEE Xplore
- Scopus
- Web of Science Core Collection
- ScienceDirect
- SpringerLink
- ACM Digital Library

The search period covered publications from January 2014 to January 2025, corresponding to the rise of deep learning-based perception systems and the increasing deployment of multimodal sensing in advanced driver assistance systems (ADAS) and higher-level autonomous vehicles.

Search strings were constructed using Boolean operators and keyword groupings that reflected three core conceptual pillars: autonomous driving, sensor modalities, and fusion methodologies. The search terms included combinations of:

- “autonomous vehicle” OR “self-driving car” OR “automated driving system”
- “sensor fusion” OR “multimodal perception”
- “LiDAR” OR “radar” OR “camera” OR “4D radar” OR “event camera” OR “GNSS” OR “IMU”
- “fusion architecture” OR “deep learning” OR “transformer” OR “BEV perception”

A representative search expression was:

(“autonomous vehicle” OR “self-driving car”) AND (“sensor fusion” OR “multimodal perception”) AND (“LiDAR” OR “radar” OR “camera”) AND (“deep learning” OR “fusion architecture”)

To improve methodological transparency, the search process was conducted iteratively in three refinement rounds. The first round focused on broad recall-oriented terms to identify dominant terminology used across autonomous driving and multimodal sensing studies. In the second round, the query set was refined using terms frequently appearing in highly cited review and benchmark papers, such as “BEV perception”, “4D radar”, “cross-attention”, and “foundation model”. The final round introduced architecture-specific descriptors related to transformer-based fusion, uncertainty-aware perception, and fail-operational safety. This iterative refinement reduced terminology bias and improved coverage of emerging subfields that may not be consistently indexed in older databases.

The search was restricted to peer-reviewed journal articles and conference proceedings published in English. In addition to database queries, backward and forward snowballing techniques were applied to highly cited publications to identify additional relevant studies not captured by keyword searches.

The study selection process strictly followed the four-stage PRISMA workflow: identification, screening, eligibility, and inclusion. The initial database search yielded 1320 records. After removing 234 duplicate entries, 1086 unique publications remained for screening. Titles and abstracts were reviewed to assess relevance with respect to multimodal fusion in autonomous driving. During this stage, 842 records were excluded because they focused on single-modality perception, non-automotive applications, or lacked technical contributions relevant to fusion architectures. A total of 244 full-text articles were assessed for eligibility. Each article was evaluated against predefined inclusion and exclusion criteria. Exclusion at this stage occurred for reasons such as insufficient methodological detail, absence of experimental validation, focus on review-only synthesis without new contributions, or application domains outside autonomous vehicles. After this rigorous assessment, 66 studies were included in the qualitative synthesis.

**Figure 1:**
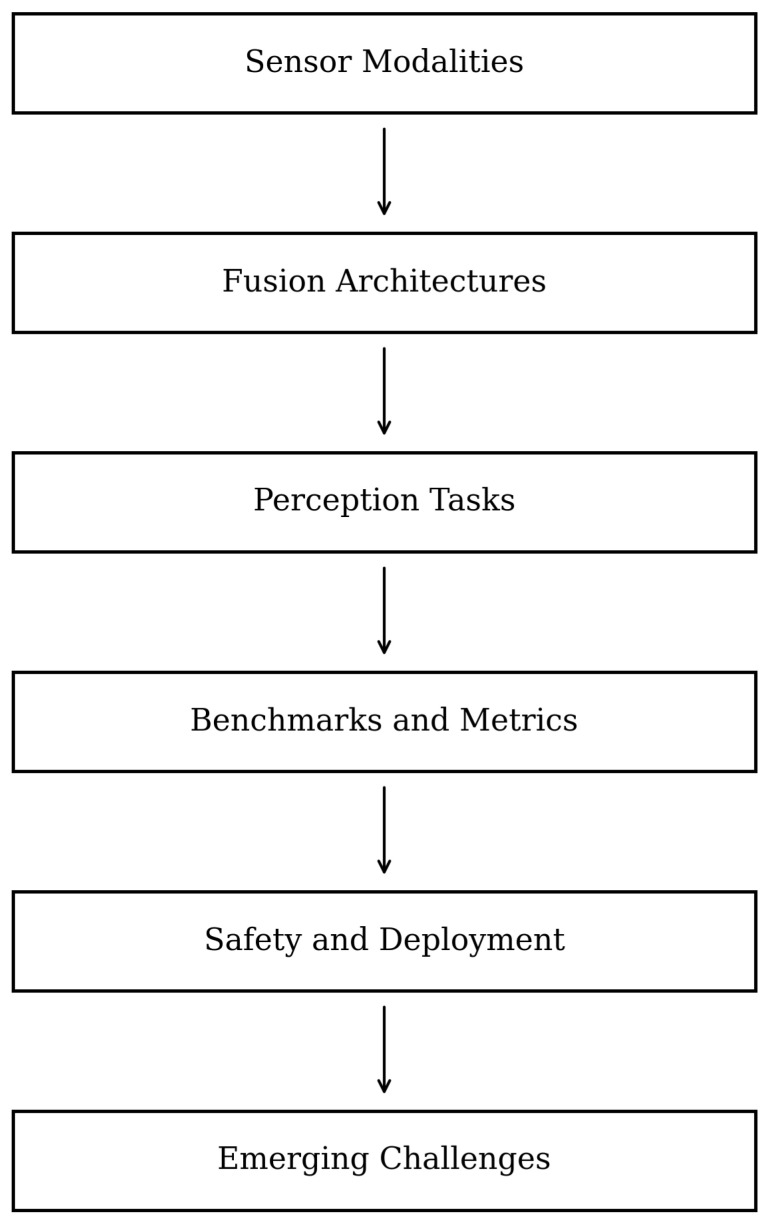
Systems-level analytical framework connecting sensor modalities, fusion architectures, perception tasks, validation benchmarks, safety constraints, and emerging research directions in autonomous vehicle multimodal perception. Source: own edited.

The PRISMA flow diagram summarizing this selection process is presented below (Figure 1).

### 2.2. Inclusion and Exclusion Criteria

To ensure methodological rigor and thematic relevance, strict eligibility criteria were applied. Studies were included if they:

- Presented original peer-reviewed research
- Explicitly addressed multimodal sensor fusion
- Focused on autonomous driving applications
- Provided quantitative experimental evaluation
- Described sensor configurations and fusion methodologies

Studies were excluded if they:

- Addressed only single-sensor perception
- Focused on non-automotive robotics
- Were editorials, commentaries, or purely conceptual
- Lacked sufficient experimental detail

To reduce subjective screening bias, the inclusion and exclusion criteria were operationalized through a structured decision matrix. During title–abstract screening, each paper was evaluated against three binary decision dimensions: (1) explicit multimodal sensing, (2) autonomous driving relevance, and (3) experimentally grounded fusion contribution. Only studies satisfying all three dimensions proceeded to full-text review. During eligibility assessment, an additional methodological sufficiency check was introduced, requiring explicit reporting of sensor configuration, validation dataset, and measurable performance indicators. This multi-stage decision framework improved consistency between reviewers and strengthened the reproducibility of the selection pipeline.

These criteria ensured that only technically robust and experimentally validated contributions formed the basis of the analysis.

A structured data extraction framework was developed to ensure consistency across included studies. For each publication, the following attributes were recorded:

- Publication year and venue
- Sensor modalities employed
- Fusion architecture category
- Learning paradigm (supervised, self-supervised, transformer-based, probabilistic, etc.)
- Benchmark validation environments and public datasets reported by the included studies
- Evaluation metrics reported
- Computational hardware platform
- Adverse condition testing
- Safety or functional validation discussion
- Reported limitations

Two reviewers independently extracted data using a standardized form. Discrepancies were resolved through discussion and consensus. This dual-review process minimized extraction bias and improved reliability.

Extracted data were subsequently coded into thematic clusters aligned with the research questions. This facilitated comparative analysis across studies.

### 2.3. Quality Assessment

To evaluate methodological robustness, each included study underwent quality assessment based on five criteria:

1. Clarity of sensor configuration description
2. Transparency of fusion architecture
3. Presence of quantitative evaluation
4. Reproducibility of experimental setup
5. Discussion of limitations and constraints

Studies were qualitatively scored and categorized into high, medium, or moderate methodological rigor groups. In addition to qualitative categorization, studies were weighted during thematic synthesis according to methodological robustness and experimental realism. Higher analytical emphasis was assigned to works validated on public large-scale benchmarks (e.g., nuScenes, Waymo, KITTI), studies including adverse-weather evaluation, and papers discussing deployment constraints or functional safety implications. This evidence-weighting approach ensured that highly cited but experimentally narrow studies did not disproportionately influence the final conclusions. Sensitivity analysis confirmed that excluding lower-quality studies did not significantly alter thematic conclusions.

Potential biases were carefully considered throughout the review process. Common risks included:

- Dataset bias (dominance of KITTI, nuScenes, or Waymo datasets)
- Positive reporting bias
- Hardware-specific performance claims
- Limited evaluation under adverse weather conditions

To mitigate bias, comparative evaluation across multiple datasets was emphasized where available. Additionally, studies were critically analyzed with respect to experimental scope and real-world applicability rather than relying solely on reported performance improvements.

### 2.4. Data Synthesis Strategy

Given the heterogeneity of experimental designs, sensor configurations, datasets, and evaluation metrics, a formal quantitative meta-analysis was not feasible. Instead, a structured qualitative synthesis approach was employed.

Studies were grouped into thematic categories:

- Sensor technology characterization
- Fusion architecture design
- Robustness and environmental evaluation
- Safety and functional validation
- Computational deployment and edge constraints
- Emerging paradigms (4D radar, neuromorphic sensing, foundation models, V2X integration)

Within each thematic cluster, cross-study comparison was performed to identify technological convergence, methodological innovation, and persistent research gaps.

The qualitative synthesis further employed a cross-thematic saturation analysis to identify repeatedly emerging architectural patterns and unresolved research bottlenecks. Themes were considered saturated when additional studies no longer introduced substantively new fusion architectures, validation approaches, or robustness strategies. This process enabled the identification of stable technological convergence trends, such as the dominance of BEV-based fusion, the growing role of transformer cross-attention, and the increasing integration of uncertainty-aware perception. It also highlighted persistent gaps, including limited real-world weather validation, explainability deficits, and insufficient hardware–software co-design analysis.

### 2.5. Methodological Limitations

Despite adherence to PRISMA standards, certain limitations remain. Restricting the review to English-language peer-reviewed publications may introduce language bias. Proprietary industrial research not publicly accessible could not be included. Furthermore, rapid advancements in foundation models and self-supervised learning may lead to publication lag relative to industry implementation. Nevertheless, the systematic design, transparent inclusion criteria, and structured synthesis process ensure that this review provides a comprehensive and methodologically sound representation of the state of the art. To enhance reproducibility, the complete search strings, screening decisions, extracted datasets, and thematic coding scheme are available upon request. The structured PRISMA-based approach allows independent replication of the review procedure. By following the PRISMA systematic review framework, this study ensures transparent study identification, rigorous screening, consistent eligibility assessment, structured data extraction, and critical synthesis. The resulting corpus of 66 carefully selected studies provides a robust and unbiased foundation for analyzing multimodal sensor fusion technologies, architectures, validation strategies, and future research directions in autonomous driving systems.

The following sections follow the proposed analytical framework by moving from the physical sensing layer toward progressively higher levels of abstraction, where sensor properties directly shape fusion design, perception performance, and system-level safety considerations.

### 2.6. Sensor Technologies in Autonomous Vehicles

Sensor types. Cameras convert incoming light into an electrical signal using CMOS- or CCD-based image sensors. RGB (monocular) cameras have become widely used in the sensor systems of autonomous vehicles due to their low cost and high spatial resolution. They typically capture color and texture information at a rate of 20–60 frames per second. However, monocular cameras do not provide direct depth information; distance must be determined using structure-from-motion methods or trained depth estimation models, which are sensitive to scale ambiguity [5,6]. Stereo cameras use two sensors placed at a known base distance, where depth can be calculated based on the disparity between corresponding pixels. According to García et al., stereo vision systems generate reliable 3D maps with improved depth perception; for example, the ZED camera has a typical base line of 12–24 cm, a field of view of 70–110°, and a frame rate of 30 Hz [7].

Event-driven or neuromorphic cameras operate asynchronously: each pixel independently reports changes in log-intensity rather than reading out entire frames. These sensors provide microsecond-level latency, a wide dynamic range, and minimal motion blur. For example, when combined with a 20 fps RGB camera, they can achieve a latency equivalent to a 5000 fps system, while requiring significantly less bandwidth [8]. Event cameras adapt excellently to dark and brightly lit environments, while enabling energy-efficient operation and precise motion detection [9,10].

In addition to cameras, radar is one of the most important sensing modalities for autonomous vehicles, especially under adverse weather and lighting conditions. Automotive radars typically use FMCW (frequency-modulated continuous-wave) technology, which allows for the simultaneous measurement of distance and relative speed using the Doppler effect. The greatest advantage of radar is that it operates reliably in rain, fog, dusty environments, and low-light conditions, where camera performance can deteriorate significantly. Although its spatial resolution is generally lower than that of cameras or LiDAR, its direct speed measurement and weather-independent robustness play a key role in multimodal sensing and redundant safety architectures. High dynamic range and low-light conditions. Automotive cameras must handle extreme lighting conditions, ranging from dark tunnels to bright sunlight. HDR (high dynamic range) sensors reduce overexposure and underexposure by combining multiple exposures, which improves detection in high-contrast scenes [11]. Larger pixel size improves sensitivity in low-light conditions, enabling nighttime object recognition [12]. Mitigating LED flicker is particularly important for the reliable recognition of LED traffic lights and taillights [13].

Advantages and limitations. The main advantage of cameras is their high spatial resolution, which enables the recognition of fine details, traffic signs, lane markings, and semantic objects. As passive sensors, they are cost-effective and energy-efficient. Their most significant limitations are: (1) the lack of direct depth information, (2) sensitivity to weather and lighting conditions, and (3) motion blur at high speeds. In contrast, radar provides lower spatial detail but complements camera systems with direct speed information and high environmental robustness. Table 1 presents a comparison of the different camera types, while the detailed technical characteristics of radar are discussed in the following subsection.

### 2.7. LiDAR Systems

LiDAR (light detection and ranging) estimates object distance by emitting laser pulses and measuring their round-trip time of flight. In automotive applications, LiDAR sensors typically operate at a wavelength of 905 nm and generate high-resolution three-dimensional point clouds with scanning rates reaching up to 200,000 points s^−1^ [3]. These sensors provide highly accurate geometric scene reconstruction, which makes them fundamental components of autonomous vehicle perception systems.

Two principal LiDAR architectures dominate current automotive applications. Mechanical rotating LiDAR systems employ a spinning assembly of laser emitters and receivers to provide a full 360° horizontal field of view. Depending on the sensor design, these systems typically contain 16–128 channels with vertical fields of view ranging from ±15° to ±45°. Their main advantages include excellent angular resolution, long detection range, and dense point cloud generation. However, their bulky construction, high cost, moving parts, and susceptibility to mechanical wear limit large-scale deployment. In addition, adverse weather conditions such as rain and fog can reduce LiDAR detection range by approximately 25% [3].

Solid-state LiDAR architectures eliminate mechanical rotation by using beam steering technologies such as micro-electro-mechanical systems (MEMS), optical phased arrays (OPA), or flash illumination. MEMS-based designs use rapidly oscillating mirrors, flash LiDAR illuminates the entire field of view simultaneously, and OPAs steer the beam electronically through phase modulation. Compared with rotating systems, solid-state LiDAR offers improved durability, lower manufacturing cost, and better suitability for automotive integration. Nevertheless, these systems often exhibit narrower fields of view and reduced point density [14]. The resulting LiDAR output is a sparse three-dimensional point cloud that encodes scene geometry with precise metric scale. Key performance parameters include detection range (commonly up to 200 m), angular resolution, point density, scanning frequency, and reflectivity sensitivity, which determines the ability to detect low-reflectance surfaces. A fundamental engineering trade-off exists between range, spatial resolution, and scan frequency, as longer detection distances generally require lower angular density or reduced update rates. Multi-return LiDAR systems further enhance environmental perception by capturing multiple reflections from semi-transparent objects such as vegetation, fences, or rain droplets [15,16,17]. LiDAR measurements are highly complementary to vision-based sensing, as they provide direct depth and geometric scale information that cameras alone cannot reliably infer. This complementary characteristic makes LiDAR one of the most important modalities in multimodal perception pipelines, particularly for 3D object detection, localization, and free-space estimation.

Automotive radar sensors complement LiDAR by providing robust range and velocity estimation under adverse environmental conditions. Frequency-modulated continuous-wave (FMCW) radar transmits chirp signals whose beat frequency encodes target distance, while Doppler shift directly measures relative velocity. Conventional radar systems estimate range, azimuth, and velocity, whereas emerging 4D imaging radar additionally resolves elevation, enabling richer spatial scene understanding. Modern 4D radar platforms achieve sub-degree azimuth resolution and detection ranges beyond 300 m, making them highly suitable for both highway and urban autonomy scenarios [10]. Their robustness to rain, fog, dust, and low-light environments makes them indispensable for fail-operational perception [18]. Ultrasonic sensors remain important for short-range obstacle detection and low-speed maneuvering tasks such as parking assistance. Operating typically in the 40–60 kHz range, these sensors are inexpensive and effective within a few meters, although they offer limited angular resolution and reduced performance at higher vehicle speeds.

Global Navigation Satellite System (GNSS) receivers provide global position estimates, but standard receivers typically offer only meter-level accuracy, which is insufficient for lane-level autonomous driving. Real-time kinematic (RTK) GNSS significantly improves this performance through correction signals and dual-frequency processing, enabling centimeter-level positioning and reliable heading estimation [19,20]. To maintain localization continuity during GNSS outages, inertial measurement units (IMUs) perform dead reckoning by integrating accelerations and angular velocities over time. Since IMU estimates drift because of sensor bias accumulation, they are commonly fused with GNSS, wheel odometry, and vehicle dynamics signals through Kalman filtering and automotive dead reckoning frameworks [18,21]. Reliable multimodal perception further depends on accurate sensor alignment and calibration. Extrinsic calibration determines the relative position and orientation between LiDAR, radar, cameras, GNSS, and IMU subsystems. Calibration may be performed offline using checkerboards, LiDAR targets, or calibration rigs, and increasingly through online self-calibration algorithms that continuously optimize sensor alignment during operation. Because calibration drift directly affects downstream multimodal fusion quality, maintaining calibration consistency throughout the vehicle life cycle remains essential. The complementary strengths of these sensing modalities provide the technological basis for the fusion architectures discussed in the following subsection.

### 2.8. Sensor Fusion Architectures

**Figure 2:**
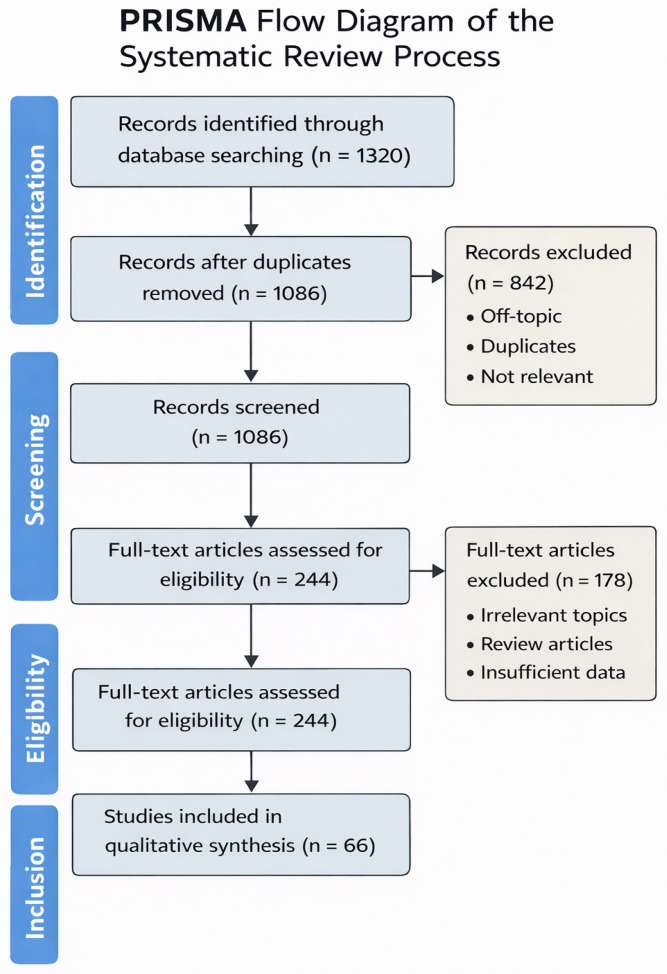
PRISMA Flow Diagram, source own edited.

Autonomous vehicles integrate multimodal sensor data using several fusion strategies that differ according to the abstraction level at which information is combined. In this review, fusion architectures are categorized into four major groups: low-level (early), mid-level (feature-level), high-level (decision-level), and transformer-based deep learning fusion. Each category offers distinct trade-offs in terms of information richness, computational efficiency, interpretability, and robustness. Figure 2 illustrates the conceptual differences among these fusion paradigms. Low-level fusion combines raw sensor measurements before feature extraction. Data from cameras, LiDARs, radars, and IMUs are first synchronized in time and transformed into a shared reference frame, such as a Bird’s Eye View (BEV) or voxel representation. This strategy preserves the maximum amount of original sensor information and enables neural networks to learn joint latent representations directly from raw multimodal signals. As a result, early fusion supports highly expressive end-to-end optimization. However, it requires precise temporal synchronization, accurate extrinsic calibration, and considerable computational resources due to the large volume of high-dimensional data. Representative examples include point-painting methods that append camera-derived semantic labels to LiDAR points, as well as BEVFusion architectures that lift multi-camera features into 3D space and combine them with LiDAR features [22]. Mid-level fusion operates on intermediate representations extracted independently from each sensing modality. Convolutional neural networks, vision transformers, or point cloud encoders first transform raw data into modality-specific latent features. These representations are then spatially aligned and fused through concatenation, summation, convolutional layers, or cross-attention mechanisms. This approach provides an effective balance between retained semantic richness and computational efficiency, making it one of the most widely adopted strategies in modern perception stacks. In BEV-based perception pipelines, for example, camera features are projected into a BEV feature space and subsequently fused with LiDAR voxel features using convolutional or transformer layers [22]. Mid-level fusion also facilitates modality-specific pretraining, transfer learning, and flexible integration of heterogeneous sensors.

High-level fusion combines the decision outputs generated by independent sensor-specific pipelines. In this architecture, each modality performs object detection, classification, or tracking separately, producing structured outputs such as bounding boxes, confidence scores, semantic labels, and motion estimates. These outputs are then merged using probabilistic decision frameworks, including Bayesian inference, Dempster–Shafer evidence theory, Kalman filtering, Joint Probabilistic Data Association (JPDA), and multi-hypothesis tracking. Compared with lower-level approaches, decision-level fusion offers superior modularity, interpretability, and fault isolation, which are especially valuable in safety-critical autonomous driving systems.

**Figure 3:**
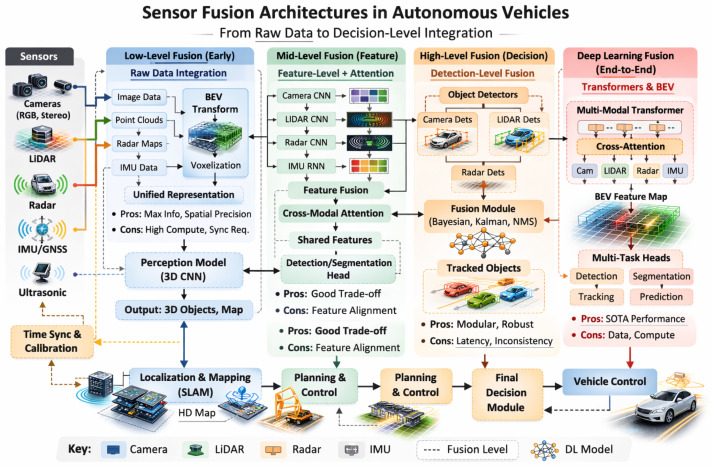
Conceptual comparison of sensor fusion paradigms in autonomous vehicles. From left to right, the figure presents low-level (early) fusion based on raw sensor integration, mid-level (feature-level) fusion combining modality-specific feature representations, high-level (decision-level) fusion merging independent detection outputs through probabilistic filtering and tracking frameworks, and transformer-based fusion using cross-attention mechanisms and Bird’s Eye View (BEV) representations. The diagram highlights the different information-processing levels, perception outputs, and decision-making pathways associated with each fusion strategy. This figure was generated for this review. source own edited.

A key strength of high-level fusion lies in its robustness to partial sensor degradation or failure. Because each sensing branch remains independently interpretable, confidence-aware reweighting and fault-tolerant fail-operational behavior can be implemented more effectively. For example, radar-based velocity estimates may remain reliable during camera degradation caused by glare, while LiDAR geometry can validate uncertain visual detections under adverse weather. This makes high-level fusion particularly suitable for object tracking, trajectory prediction, sensor redundancy validation, and safety-oriented perception pipelines aligned with ISO 26262 and SOTIF principles. Recent frameworks such as HiLO further demonstrate that uncertainty-aware decision-level fusion with transformer modules can outperform conventional feature-level fusion under varying sensor reliability conditions [23]. Transformer-based deep learning has emerged as the dominant paradigm for state-of-the-art multimodal fusion. Modern architectures employ cross-attention mechanisms to model long-range dependencies across sensing modalities while dynamically weighting sensor contributions according to spatial context and estimated reliability. For instance, BEVFusion uses transformers to project camera features into a BEV representation and fuse them with LiDAR features for unified object detection and segmentation. Self-supervised learning further extends these capabilities by exploiting large volumes of unlabeled driving data. LRS4Fusion introduces a self-supervised pretraining strategy for long-range perception that predicts future LiDAR and camera observations, improving mAP by 26.6% and extending perception range to 250 m [11]. The framework leverages sparse voxel representations and novel temporal cross-attention mechanisms to fuse multimodal features over time [24]. In addition to detection, transformer-based fusion supports joint segmentation, tracking, occupancy prediction, and multi-task learning. Nevertheless, these architectures remain constrained by substantial computational and memory requirements, as well as the need for very large-scale annotated datasets (Figure 3).

A critical emerging direction in multimodal fusion is uncertainty-aware sensor reliability modeling, where confidence estimation and dynamic sensor weighting directly influence downstream decision quality. In these frameworks, each sensing branch outputs both task predictions and confidence measures, such as epistemic uncertainty, aleatoric variance, entropy-based confidence, or evidential belief scores. These uncertainty estimates are then used to adapt fusion weights dynamically, allowing the perception stack to down-weight degraded modalities under fog, glare, occlusion, or sensor malfunction. Practical implementations commonly rely on Bayesian deep learning, Monte Carlo dropout, ensemble variance estimation, Dempster–Shafer evidence fusion, and confidence-gated transformer attention. This methodology substantially improves fail-operational behavior because the fusion process becomes reliability-adaptive rather than statically calibrated. Recent work in interaction-aware trajectory prediction further demonstrates how uncertainty-aware transformer architectures improve safety-oriented downstream planning by propagating confidence estimates into motion forecasting and risk-sensitive decision making, thereby reducing collision-prone trajectory hypotheses in complex traffic interactions [25].

A comparative synthesis of the reviewed studies indicates that no single fusion strategy consistently outperforms all others across every operational objective.

This comparative behavior can be explained by the fundamentally different design priorities of each fusion paradigm. Early fusion emphasizes information completeness, which is why it performs well in controlled conditions but struggles with computational scalability and calibration sensitivity. In contrast, mid-level fusion prioritizes representational efficiency, allowing it to achieve a strong balance between accuracy and deployability in real-world systems. High-level fusion focuses on modularity and fault isolation, which explains its superior robustness and suitability for safety-critical applications, even at the cost of reduced peak accuracy. Transformer-based fusion, by comparison, prioritizes expressive cross-modal reasoning, which leads to state-of-the-art benchmark performance but introduces significant computational overhead and challenges in interpretability and certification. Instead, performance depends strongly on the target task, sensor reliability, environmental complexity, and deployment constraints. Early fusion tends to preserve the richest raw information and can achieve strong results in tightly synchronized sensor configurations, but its computational and calibration demands limit scalability. Mid-level fusion, particularly BEV-based and transformer-enhanced architectures, most frequently delivers the highest benchmark accuracy in 3D object detection and segmentation tasks due to its balance between semantic richness and computational tractability. High-level fusion generally exhibits lower peak benchmark performance but offers superior interpretability, modularity, and fault isolation, making it especially suitable for safety-critical fail-operational pipelines and uncertainty-aware tracking systems. Transformer-based fusion currently represents the strongest performer in benchmark-driven perception tasks; however, its computational complexity, reduced explainability, and limited certifiability remain important deployment barriers. Across the reviewed literature, the most effective strategy therefore depends less on absolute benchmark superiority and more on the intended trade-off between accuracy, robustness, computational efficiency, and safety assurance. However, uncertainty propagation and certifiable interpretability remain underexplored in current transformer-based fusion frameworks.

To further clarify the comparative properties of fusion strategies, it is important to explicitly highlight the key trade-offs that influence their practical applicability. Early fusion preserves the richest raw sensor information and enables highly expressive joint representations; however, it imposes very high computational cost, strict synchronization requirements, and significant calibration sensitivity, which limit scalability in real-time automotive systems. Mid-level fusion offers a balanced compromise between performance and efficiency by combining modality-specific features, but still requires substantial computational resources and careful feature alignment. High-level fusion, in contrast, provides strong robustness and interpretability, with lower computational burden and improved fault isolation, making it particularly suitable for safety-critical and fail-operational systems; however, it may sacrifice peak perception accuracy due to limited cross-modal interaction. Transformer-based fusion achieves state-of-the-art performance by modeling complex cross-modal dependencies, but introduces very high computational and memory demands, reduced explainability, and challenges for real-time deployment on automotive-grade hardware. Overall, the choice of fusion strategy is fundamentally governed by trade-offs between computational cost, robustness under sensor degradation, interpretability, and deployment constraints, rather than by accuracy alone.

A more detailed systems-level comparison reveals that computational burden and robustness differ substantially across fusion paradigms. Early and transformer-based fusion provide the richest cross-modal interactions but impose the highest latency, memory footprint, and synchronization overhead. In contrast, high-level fusion significantly reduces computational load because each modality is processed independently, enabling lower-latency deployment and stronger fault isolation. From a robustness perspective, decision-level fusion performs best under sensor degradation and adverse weather because confidence-aware redundancy can be applied at the decision stage. Mid-level fusion offers the most balanced trade-off between perception accuracy, environmental robustness, and deployability, which explains its dominant role in practical autonomous driving stacks.

These differences highlight that the superiority of a given fusion approach is context-dependent rather than absolute: approaches that maximize accuracy under benchmark conditions are not necessarily optimal for real-time deployment, while architectures designed for robustness and fault tolerance may provide lower peak performance but significantly higher reliability in safety-critical environments.

### 2.9. Environmental Perception Tasks Enabled by Fusion

The ultimate goal of multimodal fusion is to perceive the environment accurately and support decision making. This section describes perception tasks that benefit from fusion. Object detection estimates the location, orientation and category of traffic participants in 3-D space. LiDAR provides precise depth, while cameras deliver rich texture and color cues; radar adds velocity information. Fusion improves detection accuracy, especially for distant and small objects. Detection performance is commonly measured using mean Average Precision (mAP) on bounding boxes, with Intersection over Union (IoU) thresholds defined per class. BEV-based detectors such as BEVFusion, CenterPoint and BEVFormer fuse LiDAR and camera features to achieve state-of-the-art results. High-level fusion approaches combine detections from separate camera and LiDAR networks using probabilistic filtering or non-maximum suppression. Fusion reduces false positives and improves recall for pedestrians and cyclists. The nuScenes Detection Score (NDS) aggregates mAP and other quality metrics to evaluate detection and tracking [26]. Segmentation assigns a semantic label to each pixel (or point) and distinguishes individual instances. LiDAR segmentation classifies each 3-D point into categories such as car, pedestrian or vegetation. Camera-LiDAR joint segmentation leverages camera semantics to refine LiDAR segmentation, e.g., by projecting LiDAR points onto the image and fusing features. Multimodal segmentation networks use shared backbones and cross-attention to combine pixel and point features. Instance segmentation further separates individual objects within the same class, which is important for tracking. Fusion helps differentiate occluded objects and improves segmentation of distant or small targets. Evaluation metrics include IoU and mean Intersection over Union (mIoU) across classes. Tracking maintains identities of detected objects across frames, predicting their motion and updating state estimates. Kalman filter is a classical approach for linear Gaussian motion models; it predicts state and updates with measurements. Joint Probabilistic Data Association (JPDA) associates measurements to tracks when multiple hypotheses exist. Deep SORT uses appearance embeddings from cameras combined with Kalman filtering for robust tracking. Multimodal tracking fuses detection lists from LiDAR, radar and cameras; radar velocity estimates improve motion prediction, while camera appearance features aid re-identification. Fusion reduces identity switches and improves long-term tracking in crowded scenes. Localization estimates the vehicle’s pose within a global or local map. High-definition maps provide detailed lane markings, traffic signs and 3-D landmarks; aligning perception data with HD maps improves localization accuracy. LiDAR SLAM performs simultaneous localization and mapping using LiDAR point clouds; it constructs a map and estimates pose by aligning consecutive scans. Visual–LiDAR SLAM combines visual features and LiDAR geometry, leveraging the texture information from cameras to enhance robustness in low-texture environments. GNSS/IMU data provides global position and orientation priors; fusing these with vision and LiDAR via factor graphs or extended Kalman filters mitigates drift [27]. Map-based localization is essential for urban driving and long-range planning. Free-space detection identifies drivable regions and road boundaries. Cameras capture lane markings and road surface texture; LiDAR detects curb height and obstacles; radar measures road contour under adverse conditions. Fusion improves free-space segmentation by combining camera semantics with LiDAR elevation. Drivable area detection is critical for trajectory planning and safe manoeuvres. A cross-study comparison indicates that the strongest gains are consistently observed in scenarios involving partial occlusion, distant objects, and degraded visual context, suggesting that the main scientific advantage of multimodal fusion lies in robustness-oriented redundancy rather than average-case benchmark optimization.

### 2.10. Benchmark Datasets and Evaluation Metrics

KITTI. The KITTI Vision Benchmark Suite provides stereo images, optical flow, visual odometry, 3-D object detection and tracking sequences captured using a Volkswagen Passat. The sensor suite includes a Velodyne HDL-64E LiDAR spinning at 10 Hz (~100 k points per cycle), two grayscale cameras and two color cameras (1.4 MP) triggered at 10 Hz, as well as a GPS/IMU unit [28]. KITTI covers urban, rural and highway scenes with challenging lighting conditions and provides ground-truth 3-D bounding boxes. nuScenes. nuScenes is a large-scale dataset with 1000 20-s scenes collected in Boston and Singapore. Each scene includes synchronized data from 6 cameras, 1 LiDAR, 5 radars, an IMU and GPS. The dataset contains 1.4 million camera images, 390 k LiDAR sweeps and 1.4 million 3-D bounding boxes annotated for 23 classes [29]. It also provides ego-vehicle pose, high-definition maps and weather labels. The NDS metric used in nuScenes combines mAP and attributes such as velocity error, size error, orientation error and translation error [26]. Waymo Open Dataset. Waymo’s perception dataset comprises 2030 20-s segments collected at 10 Hz across varied U.S. locations. The sensor suite includes one mid-range LiDAR, four short-range LiDARs, five high-resolution cameras and calibration data. The dataset provides 12.6 million 3-D bounding box labels and 11.8 million 2-D bounding boxes [30]. It supports 3-D detection, tracking and motion forecasting tasks and includes static and dynamic map elements (Table 2).

Evaluation metrics quantify perception performance and computational efficiency. Common metrics include: Mean Average Precision (mAP). Average precision over recall thresholds; used in 2-D and 3-D detection. nuScenes uses class-specific IoU thresholds and aggregates across classes [26]. IoU (Intersection over Union). Ratio of intersection to union between predicted and ground-truth regions; used for segmentation and detection. NDS (nuScenes Detection Score). Weighted average of mAP and mean true positive metrics, accounting for velocity error, size error, orientation error, translation error and attribute error [26].

ADE/FDE (Average Displacement Error/Final Displacement Error). Used for trajectory prediction; measures mean and final position errors between predicted and ground-truth trajectories. Latency. Time delay from sensor capture to output; real-time perception typically demands <100 ms latency [31]. Energy consumption. Power required to process sensor data; critical for edge deployment. Deep neural networks on GPUs can consume hundreds of watts [32]; specialised accelerators aim to reduce this by an order of magnitude [33].

### 2.11. Robustness in Adverse Conditions

Rain introduces scattering and refraction that degrade camera visibility and LiDAR returns. Large raindrops cause blur on lenses and produce spurious reflections. Controlled experiments show that heavy rain (45 mm h^−1^) reduces LiDAR recognition distance by about 30% and decreases the number of valid points (NPC) by 45% [7]. Radar is relatively unaffected because radio waves at 77 GHz penetrate rain; however, strong rain can introduce noise. Effective strategies include using radar as a fallback and applying neural networks trained with weather augmentation. Data augmentation approaches synthesise rain streaks and droplets on images or simulate raindrop occlusions in LiDAR point clouds. Fog comprises tiny water droplets that scatter light, severely attenuating LiDAR and camera signals. Studies report that LiDAR detection ranges decrease by roughly 25% in fog [7], with no points observed beyond 20 m when visibility drops to 50 m [8]. Radar maintains performance under fog, making radar–camera fusion essential. Domain adaptation techniques, such as style transfer and adversarial training, aim to adapt models trained on clear conditions to foggy scenarios [34,35]. Additionally, sensors can incorporate weather filters that estimate visibility from LiDAR intensity and adjust detection thresholds. Snowflakes reflect and scatter LiDAR and camera signals, creating false positives. Snow accumulation on sensor surfaces further obstructs view. Snow presents a mixed challenge for multimodal sensing systems. Camera performance is degraded by reduced contrast, snowflake occlusions, and reflections from highly reflective surfaces. LiDAR experiences backscatter and point sparsification caused by suspended snow particles, particularly during dense snowfall. Automotive radar generally maintains reliable target detection because millimeter-wave signals penetrate snow more effectively than optical modalities; however, several studies report that wet snow and high-sensitivity radar configurations may generate clutter returns or transient false positives that can be misinterpreted as obstacles [4]. This effect becomes more pronounced in urban environments where multipath reflections from metallic infrastructure and accumulated snowbanks increase measurement ambiguity [36]. Consequently, uncertainty-aware sensor fusion and temporal filtering are essential for maintaining robust object tracking in snowy conditions [17]. Fusion frameworks incorporate temporal filtering and multi-hypothesis tracking to suppress transient snow detections. Sensor heating and self-cleaning mechanisms help maintain clear optics Table 3.

Low illumination at night challenges cameras; HDR and large pixel sensors improve sensitivity [17]. LiDAR and radar remain effective at night because they emit their own energy. Event cameras exhibit excellent low-light performance due to high dynamic range [14]. Fusion algorithms must weigh sensor inputs based on reliability; for example, radar and LiDAR may dominate at night. Training data should include nocturnal scenes and long-exposure artifacts. Domain adaptation and augmentation. Robust perception under adverse conditions often requires domain adaptation. For example, the GRAMME system learns masks to filter out unreliable LiDAR and camera regions under rain and fog [9]. Data augmentation techniques synthesise rain, fog and snow to increase diversity. Generative models can create paired clear and degraded samples for supervised training. Sensor reliability modelling estimates noise variance under different weather, enabling uncertainty-aware fusion and dynamic sensor weighting. Across the reviewed studies, adverse-weather robustness emerges as a major unresolved bottleneck, particularly due to the lack of standardized cross-dataset validation protocols and uncertainty-aware evaluation metrics.

### 2.12. Safety, Redundancy and Functional Validation

ISO 26262 is an international functional safety standard for electrical and electronic systems in road vehicles. It defines a risk-based approach across the development lifecycle, from concept to decommissioning, and uses Automotive Safety Integrity Levels (ASILs) to quantify acceptable risk. PTC notes that ISO 26262 guides manufacturers to detect and mitigate hazards caused by system malfunctions and emphasises verification and validation of safety mechanisms [34]. It is not a regulation but fosters trust among stakeholders. ASIL levels (A–D) correspond to increasing severity, exposure and controllability; ASIL-D components require the most rigorous processes. Safety analysis includes failure modes and effects analysis (FMEA), fault tree analysis (FTA) and hardware/software metrics. SOTIF [31] addresses the Safety of the Intended Functionality, focusing on hazards arising from functional insufficiencies or misuse in the absence of malfunctions [37]. It complements ISO 26262 by considering limitations of perception algorithms and sensors when operating as designed. SOTIF requires identifying unknown unsafe scenarios, evaluating system performance in untested conditions (e.g., novel objects or weather), and implementing measures to mitigate risks. For sensor fusion, SOTIF motivates robustness to rare and unexpected scenarios and encourages continuous data collection and model updating [6]. Functional safety demands redundancy in both hardware and software. Vehicles often deploy dual or triple perception pipelines that process sensor data independently. For instance, one pipeline may rely on camera–LiDAR fusion, while another uses radar–camera fusion. Outputs are cross-checked, and discrepancies trigger safe responses. Redundant pipelines allow continued operation despite single faults, enabling fail-operational behavior. Fault detection mechanisms monitor sensor health (e.g., self-diagnostics, plausibility checks) and raise alerts when data becomes unreliable. For example, a LiDAR may detect a blockage if the return intensity falls below threshold; the system can then rely more heavily on radar. National Instruments (NI) explains that compute-platform validation includes verifying network interfaces, sensor interfaces under load, power consumption, thermal performance, and GNSS synchronisation [37]. They emphasise simulation and software-in-the-loop testing that covers millions of miles in the cloud to evaluate perception and sensor fusion stacks [38]. ASIL-D validation at semiconductor production ensures redundancy and identifies single points of failure [38]. Safety standards require documenting test coverage, fault injection results and safety case arguments.

Fail-operational design ensures that the system continues to operate safely after faults occur. This may involve duplicating sensors, processors and power supplies; adopting diverse algorithms to reduce common-cause failures; and implementing graceful degradation modes (e.g., reducing speed or switching to Level 2 when full autonomy fails). The redundant perception stack illustrated in Figure 3 shows three parallel pipelines feeding a fusion layer and a fault detector. When a fault is detected, the system reconfigures to rely on the remaining pipelines. ISO 26262 and SOTIF provide guidance for designing such architectures; metrics such as Fault Tolerance Time Interval (FTTI) and Diagnostic Coverage quantify how quickly faults must be detected and mitigated [39,40].

### 2.13. Computational Constraints and Edge Deployment

**Figure 4:**
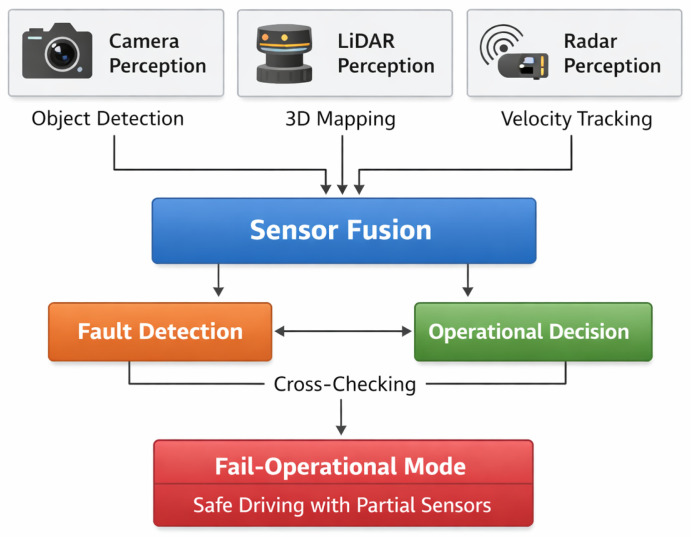
Conceptual redundant perception stack architecture. Separate pipelines for camera, LiDAR and radar process data independently and feed a fusion layer. A fault detector monitors sensor health and triggers a fail-operational mode if a pipeline fails. This figure was generated for this review. source own edited.

Autonomous vehicles must process vast amounts of data in real time. A single vehicle can produce tens of terabytes of sensor data per hour; event-based cameras reduce data volume by only reporting changes [41], yet overall computational load remains high. Perception algorithms must meet stringent latency (<100 ms) and reliability requirements [42] while operating under power and thermal constraints. Edge deployment demands hardware accelerators and efficient software architectures. Most Level 2 and early Level 3 vehicles rely on Graphics Processing Units (GPUs) for deep neural network inference due to their programmable parallel architecture. However, GPUs consume significant power (hundreds of watts) and require cooling, reducing vehicle range and increasing cost. The Edge AI and Vision Alliance notes that GPUs are not as fast or cost-effective as custom chips (ASICs) and that Level 3+ autonomy may require hundreds or thousands of watts [43]. High power consumption of GPUs, magnified by cooling requirements, can drastically degrade driving range [44] (Figure 4). To address this, the industry is developing domain-specific AI accelerators (e.g., NPUs, TPUs, FPGAs and ASICs) that deliver higher throughput per watt. Custom accelerators can provide 10× the speed of GPUs while consuming 1/10 the power [45]. These accelerators integrate neural processing units, DSPs and embedded memory within a heterogeneous System-on-Chip (SoC) architecture. Modern ADAS/ADS SoCs integrate multiple processing cores and interfaces. The Global Semiconductor Alliance describes that safe path planning requires gathering data from multiple sensors, performing signal processing and decision-making at low latency [46]. Next-generation SoCs use a heterogeneous architecture with a network-on-chip (NoC) to connect camera, radar, LiDAR and GNSS processing units [23]. High-performance DSP cores handle signal processing (e.g., Cadence Tensilica Vision processors), while multi-core CPUs and neural network processors (e.g., Cadence DNA 100) perform AI inference [47]. To deliver high bandwidth, SoCs employ memory interfaces such as LPDDR4X/5, GDDR6 and high-speed Ethernet (2.5G, 5G, 10G) [48]. Automotive SoCs must satisfy AEC-Q100 temperature and reliability standards and implement functional safety mechanisms aligned with ISO 26262 [49]. Edge computing minimises latency and preserves privacy by processing data locally. 3D InCites emphasises that cloud computing architectures hinder real-time AI due to latency and security concerns; therefore, deep learning must be integrated into edge computing frameworks [50]. Edge AI enables object detection and tracking within 3 ms with high reliability, but this requires hardware capable of trillions of operations per second. High power consumption of GPUs imposes a heavy cooling load; custom AI accelerators deliver order-of-magnitude improvements in power efficiency [6]. Edge AI chips combine CPUs, NPUs, DSPs and memory in a single package and often use advanced packaging (multi-chip modules) to optimise thermal properties [51]. Vehicular edge computing systems must deliver real-time processing, reliability, scalability and security while operating within tight energy budgets [52].

Real-time perception requires deterministic execution. MethodsX describes an edge–cloud sensor fusion system using a Raspberry Pi and Jetson Nano that achieves latency below 100 ms through hardware optimisation and watchdog timers [36]. Watchdog timers perform health checks and trigger fail-safe modes when tasks overrun, ensuring reliability. However, high-resolution inputs increase computational load; deep learning models demand significant memory and energy, limiting scalability on low-power devices [36]. Balancing model complexity with hardware capability remains a key research challenge.

### 2.14. Emerging Trends

4D imaging radar extends traditional radar by adding elevation, producing dense point clouds with range, azimuth, elevation and velocity information. NXP reports that 4D radar achieves azimuth resolution under one degree and detection ranges beyond 300 m, offering comprehensive spatial awareness in all weather conditions [10]. Aptiv explains that 4D radar identifies object height, distinguishes overhanging signs from obstacles and improves detection of road contours and boundaries [20]. Its FLR4+ radar doubles range resolution, triples vertical FOV and supports machine-learning-based elevation discrimination [21]. 4D radar’s robustness and long range make it a key sensor for Level 4–5 autonomy. Future work includes reducing cost and power consumption and integrating radar data with LiDAR and camera features in unified neural architectures. Event-based neuromorphic sensors mimic biological retinas by emitting spikes only when pixel intensities change. They provide microsecond-level temporal resolution, high dynamic range and low power consumption. Neuromorphic vision sensors can capture dynamic motion without motion blur and adapt to dark and bright stimuli [15]. IEEE researchers highlight that mimicking the retina could help achieve human-like perception; these sensors operate on principles distinct from CMOS cameras and may unlock energy-efficient perception [53]. They enable lower latency because each pixel operates independently, eliminating the need for global exposure [15]. However, processing asynchronous event streams requires specialised algorithms and hardware. Integrating neuromorphic sensors with standard cameras and LiDAR demands novel fusion strategies and event-driven neural networks. Self-supervised learning (SSL) leverages unlabeled data to learn representations by solving pretext tasks. In sensor fusion, SSL can pre-train models to predict future sensor observations, reduce dependence on labeled datasets and extend perception range. LRS4Fusion uses a self-supervised pretraining scheme that reconstructs future LiDAR points and camera frames to train a sparse voxel fusion network. The approach extends perception distances to 250 m, improves mAP by 26.6% and reduces Chamfer distance by 30.5% compared with supervised methods [11]. It employs a sparse attention mechanism to fuse camera and LiDAR features and addresses the scarcity of long-range labels by learning from unlabeled sequences [29]. SSL in autonomous driving also includes contrastive learning for visual features and self-distillation across modalities. Future research must ensure safety by preventing representation collapse and evaluating SSL models in real-world conditions. Large foundation models trained on diverse data have revolutionised natural language and computer vision. Autonomous driving is adopting foundation models to unify perception, prediction and planning. Waymo’s Foundation Model comprises a Sensor Fusion Encoder and Driving Vision-Language Model (VLM) [5]. The Sensor Fusion Encoder fuses camera, LiDAR and radar inputs over time to produce objects, semantics and rich embeddings for downstream tasks; it represents the vehicle’s “reflexes” [54]. The Driving VLM uses rich camera data and world knowledge to interpret rare or complex scenariosfor example, a vehicle on fire requiring a detour [55]. The foundation model underlies Waymo’s Driver, Simulator and Critic components; large teacher models are distilled into smaller student models to meet real-time constraints [56]. Foundation models promise improved generalisation and safety validation, but they pose challenges in data requirement, computational cost and interpretability. Vehicle-to-everything (V2X) communication enables vehicles to share sensor data with other vehicles, infrastructure and pedestrians. Aptiv and Wind River demonstrated a network V2X solution where sensors on a detecting vehicle transmit perception data over Verizon’s 5G network to another vehicle’s sensor fusion stack [57]. This cooperative perception allows vehicles to see beyond line-of-sight and improves safety by providing richer environmental information [56]. Edge computing infrastructure orchestrates data distribution and meets latency requirements for safety-critical functions [56]. Standardised APIs and cloud-mediated models enable scalable V2X deployments across manufacturers [58]. Integrating V2X with onboard sensors introduces new challenges in trust management, synchronization, and cybersecurity. Future research should develop secure fusion frameworks that weigh V2X data based on confidence and account for communication delays.

### 2.15. Open Challenges and Research Directions

Although significant progress has been made, many challenges remain:

Sensor cost vs. performance trade-off. High-performance LiDAR and 4D radar are expensive. Balancing cost with the need for redundancy and long-range perception is a major barrier for mass adoption. Solid-state LiDAR and radar chip integration may reduce cost but require advances in photonics and packaging. Uncertainty-aware fusion. Sensors produce measurements with varying noise characteristics. Modelling and propagating uncertainty through the fusion pipeline enables probabilistic decision making and improves safety. Bayesian fusion and deep probabilistic networks can estimate uncertainty but remain computationally challenging. Explainable perception. Deep fusion networks act as black boxes, raising concerns about interpretability and accountability. Developing explainable AI methods that highlight which sensors and features influenced decisions is important for certification and debugging. Certifiable AI and safety assurance. Integrating AI with functional safety standards requires certifiable models. Formal verification, runtime monitoring and fail-safe mechanisms must accompany neural networks. Frameworks like SOTIF emphasise addressing unknown unsafe scenarios; verifying neural networks under all operating conditions remains an open problem. Large-scale validation. Testing autonomous systems across millions of scenarios is infeasible on public roads. Simulation and software-in-the-loop testing provide coverage but must faithfully model sensor physics, environmental variability and rare corner cases [42]. Domain randomization and generative models can create diverse synthetic data, but transferring results to the real world is nontrivial.

Data privacy and cybersecurity. V2X communication and cloud-connected perception raise concerns about data sharing and security. Attackers could inject false sensor data or spoof GNSS signals. Secure communication protocols, cryptographic authentication and anomaly detection are essential. Scalability and energy efficiency. As sensor resolution and modality count increase, computational and energy demands grow. Future research must optimise neural architectures for edge deployment, leverage sparsity, and develop hardware–software co-design strategies. Integration with foundation models. Large models promise improved generalisation but require enormous datasets and compute. Research should explore efficient transfer learning, model compression and continual learning to adapt foundation models to new environments without catastrophic forgetting. Human–machine interaction. Even in Level 4 vehicles, human oversight may be needed. Understanding how to communicate system confidence, handover requests and limitations to passengers is critical.

### 2.16. Limitations and Developments

Although this review was conducted using a structured PRISMA-based systematic methodology and includes a broad cross-section of peer-reviewed literature, several limitations must be acknowledged to contextualize the scope and interpretability of the findings.

First, the review was restricted to peer-reviewed journal articles and conference proceedings published in English between 2014 and January 2025. While this timeframe captures the most dynamic phase of deep learning-based multimodal sensor fusion development, it inevitably excludes earlier foundational works and very recent publications that may have appeared after the search cut-off date. Given the extremely rapid evolution of autonomous driving technologiesparticularly in areas such as foundation models, large-scale self-supervised learning, and 4D radarthere is a possibility of publication lag bias. Industrial breakthroughs often precede academic publications, meaning that some state-of-the-art proprietary developments are not represented in the analyzed corpus. Additionally, restricting the review to English-language publications introduces potential language bias. Relevant contributions published in other languages, particularly from rapidly developing research ecosystems in Asia, may not have been included. Publication bias is another concern. Studies reporting significant performance improvements or novel architectures are more likely to be published than those reporting negative or inconclusive results. Consequently, the literature may overrepresent successful fusion strategies and underrepresent failed implementations or deployment challenges.

A notable limitation identified during the synthesis is the strong dependence of the field on a small number of benchmark datasets, primarily KITTI, nuScenes, and Waymo Open Dataset. While these datasets are high quality and widely accepted, their dominance introduces structural bias. Many reported performance improvements are incremental optimizations specific to these datasets rather than generalizable advances validated under diverse real-world conditions. Real-world deployment environments often involve weather variability, sensor degradation, calibration drift, infrastructure differences, and long-tail scenarios that are not sufficiently represented in benchmark datasets. As a result, reported metrics such as mAP, IoU, or NDS may not directly translate into operational reliability in production autonomous vehicles. Furthermore, there is limited standardization in reporting computational performance metrics such as latency, energy consumption, or hardware resource utilization. Some studies evaluate models on high-end GPU platforms without addressing embedded or automotive-grade deployment constraints. This heterogeneity complicates direct cross-study comparison.

Another methodological limitation arises from the diversity of fusion architectures and evaluation protocols. Early fusion, mid-level fusion, late fusion, transformer-based fusion, probabilistic frameworks, and hybrid systems differ substantially in their assumptions, preprocessing pipelines, synchronization strategies, and training regimes. Because of this heterogeneity, conducting a quantitative meta-analysis was not feasible. The review therefore relies on qualitative synthesis rather than statistical aggregation of performance metrics. Moreover, many studies do not provide full architectural transparency or release code and trained models. Limited reproducibility hinders independent validation of claims and may affect the robustness of comparative conclusions.

Although this review places strong emphasis on ISO 26262 and SOTIF frameworks, it must be noted that the majority of analyzed academic works do not provide detailed functional safety validation. Many studies focus primarily on perception performance metrics without systematically evaluating:

- Fault tolerance under sensor degradation
- Redundant pipeline cross-checking
- Failure mode analysis
- Cybersecurity resilience
- Long-term operational robustness

Therefore, while technological advancements are extensively documented, comprehensive safety case integration remains underrepresented in the literature. This limits the ability to draw definitive conclusions regarding certifiable deployment readiness.

Although adverse weather conditions such as rain, fog, and snow are discussed in multiple publications, systematic evaluation under controlled environmental variability remains limited. Many robustness studies simulate adverse conditions synthetically rather than collecting real-world data under controlled meteorological scenarios. Sensor contamination, aging effects, mechanical vibration, and long-term thermal stress are rarely considered in academic evaluations. As a result, real-world degradation mechanisms may be underestimated in the current research landscape.

The domain of autonomous vehicle perception is evolving at an unprecedented pace. Emerging paradigms such as:

- 4D imaging radar
- Neuromorphic event-based sensing
- Self-supervised long-range perception
- Foundation models for driving
- V2X cooperative perception

are currently in transition from research prototypes to scalable implementations. Consequently, the conclusions drawn in this review represent a snapshot of the field rather than a static assessment. Certain architectural trends may evolve rapidly as hardware accelerators, sensor miniaturization, and AI model compression techniques advance. A significant limitation stems from the gap between academic research and industrial deployment. Automotive OEMs and technology companies (e.g., Waymo, Tesla, Mobileye, NVIDIA, Aptiv) often develop proprietary sensor fusion frameworks, safety validation pipelines, and data infrastructures that are not publicly disclosed. Therefore, some of the most advanced real-world systems may not be reflected in peer-reviewed publications. This asymmetry between academic transparency and industrial confidentiality constrains the completeness of any systematic review.

Reported performance metrics frequently depend on the specific computational platform used. High-end GPUs, TPUs, custom ASICs, and automotive-grade SoCs exhibit widely varying throughput and energy profiles. Because hardware specifications are not consistently standardized across studies, comparative assessment of energy efficiency and real-time capability remains partially constrained. Additionally, many works evaluate models in offline settings rather than in closed-loop vehicle control scenarios, limiting the interpretability of latency and reliability results under real-time constraints. Due to the high variability in experimental setups, sensor configurations, evaluation metrics, and performance reporting standards, a statistical meta-analysis was not feasible. Consequently, findings are based on qualitative thematic synthesis rather than pooled quantitative effect sizes. While this approach provides structured insight into trends and gaps, it limits the statistical generalizability of performance comparisons.

Despite these constraints, the systematic PRISMA-based methodology ensures transparency and reproducibility of the selection process. The identified 66 studies represent a carefully curated and methodologically screened corpus that captures the dominant architectural paradigms, technological innovations, and validation practices within multimodal sensor fusion research.

However, readers should interpret conclusions with awareness of:

- Dataset-centric evaluation bias
- Limited real-world robustness validation
- Hardware-dependent performance variability
- Underrepresentation of proprietary industrial solutions
- Rapid technological evolution in AI-driven perception

Future systematic reviews may benefit from including industrial white papers (where accessible), multilingual database expansion, standardized computational reporting frameworks, and scenario-based robustness benchmarking. A key unresolved hypothesis is whether uncertainty-aware transformer fusion can satisfy real-time fail-operational constraints in Level 4 deployment.

## 3. Discussion

The results of this systematic review clearly demonstrate that multimodal sensor fusion has evolved from a performance-enhancing technique into a structural prerequisite for higher levels of driving automation. While early autonomous systems relied on limited sensor configurationstypically camera–radar combinationsthe current trajectory of research and development indicates that robust perception requires a tightly integrated, multi-layered sensing ecosystem. This ecosystem must simultaneously satisfy geometric accuracy, semantic richness, environmental robustness, computational feasibility, and functional safety constraints. The discussion therefore extends beyond comparing sensor modalities and instead examines how fusion architectures reshape the entire perception paradigm of autonomous vehicles.

One of the most fundamental observations emerging from the literature is that the complementarity of sensors is inseparable from system-level redundancy. Cameras provide dense semantic information and high-resolution texture but lack intrinsic depth estimation and are sensitive to illumination variability. LiDAR supplies precise three-dimensional geometry yet degrades under fog, heavy rain, or snow. Radar contributes direct velocity measurements and superior weather robustness but offers lower angular resolution and susceptibility to multipath artifacts. GNSS and IMU systems provide global and inertial positioning but suffer from drift and signal obstruction. No modality independently achieves reliable operation across all operational design domains. Consequently, fusion is not merely an accuracy optimization strategy but a reliability architecture that compensates for modality-specific weaknesses. The reviewed studies suggest that early fusion approaches focus primarily on exploiting complementarity by integrating raw data into unified representations such as Bird’s Eye View (BEV) maps. Mid-level fusion strategies align intermediate features extracted independently from each modality, while high-level fusion combines detection outputs probabilistically. More recently, transformer-based architectures have become dominant, offering unified cross-modal attention mechanisms capable of implicitly learning sensor reliability weighting. This shift toward attention-driven fusion represents a paradigm change: instead of manually designing modality hierarchies, the network learns context-dependent sensor importance. However, this architectural unification also introduces new challenges in computational cost, interpretability, and safety certification. The dominance of deep learning–based fusion models reflects a broader transition from modular engineering pipelines to data-driven perception systems. Transformer-based BEV frameworks demonstrate remarkable performance gains in detection and segmentation benchmarks. Yet the growing architectural complexity highlights a tension between performance optimization and deployment feasibility. Automotive-grade hardware platforms operate under strict latency (<100 ms), energy, and thermal constraints. High-capacity models that achieve state-of-the-art benchmark results may not be directly transferable to embedded environments without model compression, pruning, quantization, or hardware-specific acceleration. Therefore, the evolution of fusion architectures must be accompanied by hardware–software co-design principles to ensure scalability beyond research prototypes.

Another significant insight concerns dataset dependence and generalization. The majority of studies rely heavily on benchmark datasets such as KITTI, nuScenes, and Waymo Open Dataset. While these datasets provide standardized evaluation protocols, they do not fully capture the diversity of real-world operational environments. Weather variability, sensor aging, regional infrastructure differences, and long-tail corner cases remain underrepresented. Consequently, incremental improvements in metrics such as mAP or NDS do not necessarily equate to improved operational safety in deployment contexts. The field risks optimizing toward benchmark saturation rather than real-world robustness. Bridging this gap requires more comprehensive validation strategies, including long-duration field testing, sensor degradation modeling, and cross-climate scenario evaluation. Environmental robustness remains one of the most persistent challenges. Empirical findings consistently show that rain, fog, and snow significantly degrade camera and LiDAR performance. Radar mitigates some of these limitations but introduces its own spatial ambiguities. Fusion improves resilience by leveraging modality diversity; however, most fusion frameworks still prioritize deterministic accuracy metrics over uncertainty quantification. From a safety perspective, modeling uncertainty and confidence propagation is as critical as maximizing detection rates. Probabilistic fusion methods and uncertainty-aware neural architectures therefore represent essential directions for future research. Without explicit reliability estimation, even high-performing models may fail unpredictably under distribution shifts.

The integration of functional safety standards, particularly ISO 26262 and SOTIF, further reframes sensor fusion as a safety-driven architectural requirement. Redundant perception pipelines enable cross-validation of sensor outputs and facilitate fail-operational strategies. Nevertheless, most academic works focus on perception accuracy without explicitly embedding safety case traceability or fault-tolerance analysis. This indicates a disconnect between research innovation and automotive certification practice. For multimodal fusion to achieve industrial maturity, it must incorporate runtime monitoring, fault detection mechanisms, structured logging, and explainability features that align with formal safety validation processes. Computational scalability is another defining constraint shaping future development. As sensor resolution increases and additional modalitiessuch as 4D radar or event-based camerasare integrated, data throughput escalates dramatically. Traditional GPU-based architectures face limitations in power efficiency and thermal dissipation, particularly for Level 4–5 vehicles requiring continuous high-performance inference. The emergence of dedicated AI accelerators and heterogeneous automotive SoCs suggests a shift toward domain-specific compute platforms. Sparse representations, event-driven processing, and edge-centric architectures will likely play a pivotal role in balancing performance with energy efficiency.

Emerging sensing technologies further complicate and enrich the fusion landscape. 4D imaging radar narrows the performance gap between radar and LiDAR by adding elevation resolution and dense point-cloud capabilities, while maintaining robustness under adverse weather. Neuromorphic event-based sensors promise ultra-low latency and high dynamic range, potentially improving high-speed perception in challenging lighting conditions. Foundation models introduce a more radical conceptual shift by attempting to unify perception, reasoning, and prediction within large-scale pre-trained architectures. While these developments signal substantial progress, they also intensify concerns regarding computational cost, interpretability, and certifiability. Cooperative perception through V2X communication extends the sensing horizon beyond line-of-sight constraints and offers potential mitigation for occlusion-related failures. However, incorporating external sensor data introduces additional uncertainties related to communication latency, trustworthiness, and cybersecurity. Secure, delay-aware, and confidence-weighted cooperative fusion frameworks are therefore necessary to ensure that extended perception enhances rather than destabilizes system reliability. Overall, the literature converges toward a holistic understanding of multimodal fusion as a systems-level optimization challenge. Detection accuracy, robustness, safety compliance, computational efficiency, and economic feasibility cannot be addressed independently. Improvements in one dimension often introduce trade-offs in others. For example, increasing redundancy improves safety but increases cost and power consumption; adopting large transformer models enhances semantic reasoning but complicates verification and real-time deployment. The current state of research therefore represents a transitional phase. Multimodal fusion technologies have achieved impressive benchmark performance and architectural sophistication, yet the path toward scalable, certifiable, and economically viable deployment remains complex. The next generation of research must prioritize uncertainty-aware modeling, explainable cross-modal reasoning, real-world robustness validation, and integrated hardware–software design frameworks.

In conclusion, multimodal sensor fusion is not merely a technological trend but the foundational mechanism enabling higher levels of driving automation. Its future success will depend on interdisciplinary convergence across sensing physics, machine learning, safety engineering, embedded systems design, and regulatory compliance. Only through such coordinated development can autonomous perception systems transition from experimental prototypes to reliable components of intelligent transport systems.

### 3.1. Practical Recommendations and Research Guidelines

Based on the comparative synthesis of the reviewed studies, several practical and scientific recommendations can be formulated for future multimodal perception research in autonomous vehicles. First, future fusion architectures should explicitly incorporate uncertainty-aware reasoning and confidence propagation mechanisms. Current deterministic benchmark optimization strategies are insufficient for safety-critical deployment under rare corner cases and environmental distribution shifts. Second, robustness evaluation should move beyond standard clear-weather benchmarks toward standardized cross-dataset protocols involving rain, fog, snow, glare, and low-light scenarios. This would improve the external validity of multimodal fusion claims. Third, safety-oriented design principles should be integrated earlier into fusion architecture development. Functional safety standards such as ISO 26262 and SOTIF should not be treated as post hoc validation layers, but as architectural design constraints from the earliest development stages. Fourth, future systems should prioritize hardware–software co-design to ensure real-time deployment feasibility. Efficient transformer variants, sparsity-aware processing, and dedicated automotive AI accelerators are likely to become essential for scalable Level 4–5 perception systems. Finally, greater emphasis should be placed on cross-dataset reproducibility, explainable cross-modal reasoning, and large-scale real-world validation, allowing research prototypes to transition into certifiable industrial perception stacks.

### 3.2. Critical Barriers to Real-World Deployment of Emerging Paradigms

Despite the rapid emergence of foundation models, self-supervised learning, and 4D radar sensing, several critical barriers continue to limit their real-world deployment in safety-critical autonomous driving systems. First, foundation-model-based multimodal perception remains constrained by limited explainability, extremely high computational demand, and the lack of certifiable behavior under rare corner cases, which complicates ISO 26262 and SOTIF compliance. Second, self-supervised long-range fusion methods strongly depend on large-scale domain-consistent pretraining corpora, making cross-region and cross-weather generalization difficult in real-world fleet deployment. Third, although 4D radar substantially improves robustness in adverse weather, unresolved challenges remain in sparse elevation ambiguity, ghost target suppression, multipath interference, and the absence of standardized large-scale public benchmarks.

Additional barriers include online calibration drift, hardware thermal constraints, memory footprint on automotive-grade edge accelerators, and the difficulty of propagating uncertainty estimates into downstream planning modules. These limitations indicate that the primary research bottleneck is no longer benchmark-level perception accuracy, but certifiable, computationally efficient, and domain-robust deployment at fleet scale (Table 4).

## 4. Conclusions

The rapid evolution of autonomous vehicle technologies has established environmental perception as one of the most critical foundations of intelligent transportation systems. This review aimed to provide a systematic and technically grounded synthesis of the sensing modalities, multimodal fusion architectures, robustness considerations, and deployment constraints that define modern autonomous driving perception. The literature consistently confirms that no single sensing modality can ensure reliable operation across the full spectrum of real-world conditions. Cameras, LiDAR, radar, ultrasonic sensors, and GNSS/IMU-based localization systems each contribute complementary strengths while remaining vulnerable to modality-specific limitations. Consequently, multimodal sensor fusion has emerged as one of the most effective architectural strategies for achieving redundancy, robustness, and fail-operational perception in higher levels of driving automation. A major trend identified in the reviewed studies is the transition from modular perception pipelines toward unified deep learning and transformer-based fusion frameworks. Shared Bird’s Eye View representations and cross-modal attention mechanisms have significantly improved detection, segmentation, tracking, and localization performance. At the same time, these increasingly complex architectures raise important challenges related to interpretability, computational scalability, and certifiable deployment in real-time automotive environments. The review further highlights that robustness under adverse environmental conditions remains a decisive research challenge. Rain, fog, snow, glare, and low-light scenarios continue to expose the limitations of individual sensing modalities. While multimodal fusion improves resilience by dynamically leveraging complementary sensor reliability, the explicit treatment of uncertainty, confidence propagation, and corner-case awareness remains insufficiently addressed in many current frameworks. Future architectures must therefore integrate uncertainty-aware reasoning as a core design principle rather than a secondary enhancement.

Another key finding is the growing importance of functional safety and validation standards, particularly ISO 26262 and SOTIF, in shaping the design of autonomous perception systems. Redundant sensing pipelines, cross-validation mechanisms, runtime diagnostics, and fail-operational architectures are increasingly necessary to bridge the gap between academic benchmark performance and automotive-grade deployment requirements. This indicates that future progress in the field will depend as much on explainability, safety engineering, and validation methodology as on improvements in raw detection accuracy. Computational efficiency and energy-aware deployment also remain central constraints. As sensing stacks expand with higher-resolution LiDAR, 4D radar, neuromorphic cameras, and cooperative V2X inputs, the resulting data throughput increasingly demands hardware–software co-design, efficient neural architectures, sparsity-aware processing, and dedicated automotive AI accelerators. The ability to reconcile perception accuracy with real-time edge deployment will remain a defining challenge for practical Level 4–5 systems. Emerging directions such as 4D radar, self-supervised long-range perception, foundation models, and cooperative sensing suggest that multimodal fusion is entering a new phase of technological maturity. However, scalable real-world adoption will require holistic co-optimization of sensing, computation, safety, explainability, and economic feasibility. Ultimately, the future of autonomous perception depends on interdisciplinary integration across sensor physics, machine learning, embedded systems, safety engineering, and regulatory validation, enabling multimodal fusion to evolve from high-performing research prototypes into dependable components of next-generation intelligent mobility systems.

## Figures and Tables

**Table 1:** Comparison of camera systems for autonomous driving, source: own edited.

Sensor Type	Depth Information	Strengths	Limitations
RGB camera	None (monocular)	High spatial resolution; color/texture information; low cost	Requires depth inference; sensitive to lighting and weather; motion blur
Stereo camera	Disparity yields depth	Depth estimation without LiDAR; better geometry awareness than monocular	Baseline limits depth accuracy; increased cost and calibration complexity; limited low-light performance
Event camera	Encodes temporal contrast; can infer motion and depth via neuromorphic algorithms	Microsecond latency; high dynamic range; low power consumption	Generates asynchronous event streams requiring specialised processing; lower spatial resolution; limited adoption

**Table 2:** Comparative evaluation of fusion strategies, source: own edited.

Fusion Strategy	Accuracy	Robustness	Compute Cost	Interpretability	Safety Suitability
Early	High	Medium	Very high	Low	Medium
Mid-level	Very high	High	High	Medium	High
High-level	Medium	Very high	Medium	Very high	Very high
Transformer	Highest	High	Very high	Low	Medium

**Table 3:** Overview of public multimodal perception datasets, source: own edited.

Dataset	Sensor Suite	Size and Duration	Key Features
KITTI	2 color cameras, 2 grayscale cameras, Velodyne 64-channel LiDAR, GPS/IMU	≈11 k images with synchronous LiDAR scans; collected in Karlsruhe	Early benchmark; provides stereo, optical flow, 3D object detection and tracking; limited sensor diversity
nuScenes	6 cameras, 1 LiDAR, 5 radars, IMU, GPS	1000 scenes of 20 s (≈300 k frames); 1.4 M images	Diverse urban scenes in Boston/Singapore; includes radar and full sensor suite; uses NDS metric
Waymo Open Dataset	5 cameras, 1 mid-range and 4 short-range LiDARs	2030 scenes of 20 s; 12.6 M 3D boxes	High-resolution sensors and dense labeling; provides map and motion forecasting tasks

**Table 4:** Critical deployment barriers of emerging multimodal perception paradigms, source: own edited.

Technology	Main Advantage	Critical Deployment Barrier
Foundation models	Cross-task generalization	Explainability, certification, compute
Self-supervised fusion	Reduced annotation cost	Domain shift, weather transfer
4D radar	Adverse-weather robustness	Ghost targets, sparse benchmarks
Cooperative V2X	Extended perception horizon	Communication latency, trust

## Data Availability

Data sharing is not applicable. No new data were created or analyzed in this study.

## Abbreviations

The following abbreviations are used in this manuscript:

AI	Artificial Intelligence
CNN	Convolutional Neural Network
LLM	Large Language Model
RAG	Retrieval-Augmented Generation
ViT	Vision Transformer
SAM	Segment Anything Model
NLU	Natural Language Understanding
NLG	Natural Language Generation
GDPR	General Data Protection Regulation
ISIC	International Skin Imaging Collaboration
